# Wearable robotic device for upper limb recovery and predictive algorithms to emPOWER the rehabilitation of stroke survivors: a protocol of safety and RCT study (POWER)

**DOI:** 10.3389/fneur.2026.1858025

**Published:** 2026-09-14

**Authors:** Domenico Intiso, Ali Khalilianmotamed Bonab, Antonello Marco Centra, Michela Falcone, Giuseppe Guerra, Angela La Forgia, Serena Filoni, Filomena Di Rienzo, Antonio Frisoli, Domenico Chiaradia, Francesco Giuliani

**Affiliations:** 1 Unit of Neurorehabilitation, IRCCS Casa Sollievo della Sofferenza, San Giovanni Rotondo, Italy; 2 Sant’Anna School of Advanced Study, Institute of Mechanical Intelligence, Pisa, Italy; 3 Innovation & Research Unit, Fondazione IRCCS "Casa Sollievo della Sofferenza", San Giovanni Rotondo, Italy; 4 Exprivia s.p.a, Barletta, Italy

**Keywords:** artificial intelligence, robot-assisted therapy, stroke rehabilitation, upper limb recovery, usability, wearable robotics

## Abstract

**Introduction:**

Stroke remains a leading cause of long-term disability worldwide, with persistent upper limb (UL) impairment affecting approximately half of survivors. Robotic rehabilitation has shown efficacy comparable to conventional therapy, but most available devices are bulky, non-wearable, and limited to institutional settings. Wearable robotic solutions combined with artificial intelligence (AI) may overcome these barriers. The present protocol study will investigate the wearability and acceptance of a new soft, exosuit robotic device, the effect on the rehabilitation of paretic UL in stroke subjects and the role of AI for movement classification and actuation.

**Method:**

The study will be conducted in a single neurorehabilitation center and divided into 2 phases: phase A including 10 adult healthy subjects and 10 stroke survivors who will wear the experimental robot device. The usability and acceptance of device will be ascertained by System Usability Scale (SUS); User Experience Questionnaire (UEQ); Technology Assisted Rehabilitation Patient Perception Questionnaire (TARPP-Q) measures and will be considered successfully completed whether SUS and UEQ exceed 68 and 0.8 score, respectively. Phase B is a randomized controlled trial comparing wearable robotic rehabilitation with conventional therapy in stroke survivors. Neurological evaluation will be executed by the National Institutes of Health Stroke Scale (NIHSS). Functional evaluation will be quantified according to International Classification of Functioning, Disability and Health (ICF) domains by Fugl Meyer Assessment Scale-Upper extremity (FMA-UE); Motricity Index; modified Ashworth Scale; Numerical Rating Scale; Box & Block Test; Frenchay Activity Index. Functional measures will be taken at baseline, at 2 weeks, at 4 weeks, at discharge, and at follow-up, except FMA-UE. AI algorithms will be developed to automatically assess movement quality and predict assistance intensity.

**Discussion:**

The protocol tests a novel wearable device for the recovery of UL in stroke survivors overcoming the limitations of currently used robot equipment and promoting a new approach to rehabilitation outside the institutional setting. AI associated to robot rehabilitation could enhance the patient’s recovery and open the way to novel therapeutic practices.

## 1 Introduction

Stroke remains one of the leading causes of disability worldwide despite a progressive reduction in mortality over recent decades. According to global epidemiological data, stroke accounts for approximately 6.2 million deaths annually and represents the second leading cause of death globally (1). In high-income countries, stroke is the primary cause of long-term disability in adults, imposing a substantial social and economic burden on healthcare systems. Although survival rates have improved, functional recovery remains incomplete in the majority of patients: only about one-third of stroke survivors achieve full functional recovery, while 35–40% experience severe residual disability and a markedly reduced quality of life (2).

In this context, it is extremely important not only to research effective therapies to minimize brain damage but especially rehabilitative strategies capable of reducing functional limitations and promoting recovery and participation of such subjects. It is well known that stroke survivors show sensory and motor disorders involving the upper and lower limbs, requiring appropriate rehabilitative interventions. However, recovery is not the same for both limbs. Scientific literature documents that recovery of the lower limb is greater than that of the upper limb (UL) (3). After 3–6 months from the event, 55–75% of stroke subjects show functional limitations of the UL (4, 5). The functionality of the lower limb is essential for standing and walking as walking ability has morbidity and mortality implications, but the UL is involved in many essential activities of daily living (ADL), from personal care to social interaction.

Over the past two decades, several rehabilitative approaches have been developed to enhance UL recovery after stroke, including task-oriented training, constraint-induced movement therapy, neuromodulation techniques, and robotic rehabilitation (6). Among these, robot-assisted rehabilitation (RR) has gained increasing attention due to its ability to deliver intensive, repetitive, and task-specific training. Multiple systematic reviews and meta-analyses have shown that RR is at least as effective as conventional therapy in improving motor impairment and strength of the paretic UL (7–10). Nevertheless, its impact on functional use of the arm in daily life and on participation outcomes remains controversial. Furthermore, one of the main limitations of current RR approaches lies in the characteristics of currently available devices. Most robotic systems for UL rehabilitation are fixed, bulky, and confined to specialized clinical environments. Despite being occasionally described as “portable,” many existing devices remain heavy, cumbersome, and poorly adaptable to real-world contexts, limiting their applicability beyond institutional settings. They often require complex setup procedures, long donning and doffing times, and continuous supervision, which may reduce usability and acceptance by both patients and therapists (11).

Recent technological advances have fostered the development of soft, lightweight, and wearable robotic devices, aiming to overcome these limitations and support functional movements in more ecological contexts (12–15). However, evidence regarding their usability, safety, and acceptability in stroke populations remains scarce. In parallel, artificial intelligence (AI) and machine learning techniques are increasingly being explored in neurorehabilitation, particularly in patients with Parkinson’s disease and stroke to enhance assessment, monitoring, and personalization of treatment (16–18). In the context of robotic rehabilitation, AI holds promise for analyzing complex kinematic and dynamic data, identifying movement patterns, detecting compensatory strategies, and predicting recovery trajectories.

The present study protocol (POWER) aims to address two key questions in current stroke rehabilitation research. First, it seeks to evaluate the feasibility, usability, acceptability, and safety of a novel soft wearable exoskeleton for UL rehabilitation in both healthy individuals and stroke survivors (Phase A). Second, it aims to assess the effectiveness of this device in improving UL functional recovery compared with conventional therapy through a randomized controlled trial (Phase B). Additionally, the study explores the integration of AI algorithms to support automatic movement evaluation and personalized assistance modulation, with the ultimate goal of enhancing functional recovery and facilitating future home-based rehabilitation strategies.

## 2 Objectives

*Primary objectives of the study*: (a) To evaluate the use of a wearable exosuit device by healthy volunteers and ischemic stroke survivors regarding its usability, acceptability, and wearability; (b) to investigate the effectiveness of the new robotic device for functional recovery of the UL in stroke survivors.

*Secondary objective*: To verify to what extent an AI algorithm, trained on arm movements performed by healthy volunteers, can determine the correct intensity of assistance that the exoskeleton must provide to each single patient to achieve movements that can be as close as possible to those performed by the healthy volunteers.

## 3 Methods and analysis

### 3.1 Study design and setting

The study is conducted in a single center and will be executed in a neurorehabilitation setting. It is divided into 2 phases defined below as “Phase A” and “Phase B.” Due to the novelty and unique characteristic of wearable exosuit, this staged investigation design consistently adopts the principles of Regulation (EU) 2017/745 (MDR), ISO 14155 and IEC 62366-1. The first Phase (A) is conceived as an iterative feasibility and usability study aimed at optimizing the intervention protocol with investigational robotic device. We plan that iterative refinements will be necessary until progression criteria reported in the following are achieved. Only after successful completion of these predefined criteria, the study will be allowed to proceed to Phase B, which is designed to evaluate the clinical performance and effectiveness of the optimized intervention (Figure 1). Phase A will last 2 weeks. Phase B consists of a randomized controlled clinical trial (RCT) aimed at investigating the effectiveness of rehabilitation carried out using the experimental robotic device compared to rehabilitation carried out without its support. The RCT results will be reported following the Consolidated Standards of Reporting Trials (CONSORT) statement and checked with the CONSORT 2010 checklist (19).

**Figure 1:**
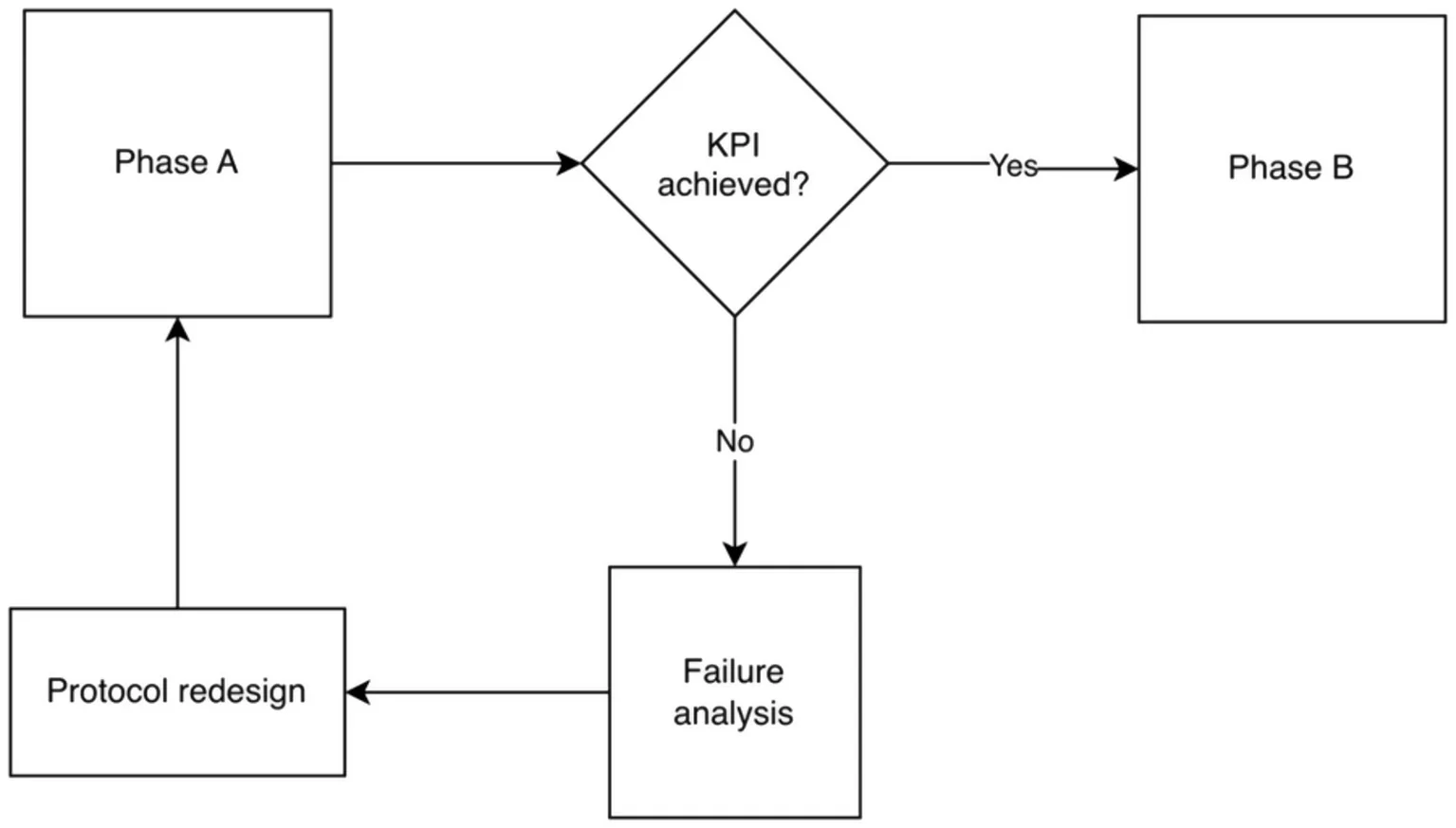
Iterative study design adopted for the clinical investigation. Phase A consists of repeated cycles of KPI (safety, usability, and feasibility) assessment. Progression to Phase B occurs only after predefined KPI values achievement. If the progression criteria are not met, a failure analysis is performed to identify limiting factors, followed by protocol redesign and a new iteration of Phase A. KPI, key performance indicator.

### 3.2 Endpoints

*Phase A—primary endpoint*: Positive evaluation by healthy subjects and stroke survivors regarding the usability, acceptability, and wearability of the exoskeletal device.

*Secondary endpoint*: Ability of an AI algorithm to assess the correctness of movements performed by the stroke participants during the use of the exoskeleton and support correct assistance intensity.

*Phase B—primary endpoint*: Superiority of the experimental wearable robotic device in recovering the functionality of the paretic UL in subjects with ischemic stroke compared to traditional rehabilitative treatment.

## 4 Population

### 4.1 Phase A—participants and procedures

The study involves the use of a wearable exoskeletal device by a cohort of 10 healthy volunteers (HVs) according to the following inclusion criteria: adult healthy subjects with age between 18 and 85 years. HVs with previous neurological diseases, orthopedic disorders involving upper limb and neoplasms will be excluded. Profession and potential experience in the rehabilitation sector will be collected; HVs will be required to wear for both dominant and non-dominant arm the robotic device and perform, under the guidance of a physiotherapist, simple flexion or extension movements of the arm, during which dynamic and kinematic variables will be measured. In addition, HVs will perform 2 “goal-oriented” gestures described below.

- The subject, seated at a table with the elbow resting on the table surface, must touch with the hand a point drawn on a board placed about 30 cm in front of them (touching).
- The subject, seated at a table with the elbow resting on the same surface, must take a glass placed in front of them (reaching).

In phase A of the study, 10 stroke survivors in the sub-acute phase will also be recruited and will undergo the same procedures as HVs. These subjects will be identified among those admitted to the rehabilitation setting of IRCCS “Casa Sollievo della Sofferenza” according to the inclusion/exclusion criteria described in Phase B. HVs will be homogeneous in demographic characteristics with respect to stroke survivors and will be recruited among healthy staff working in different departments of the hospital. To avoid age imbalances, HVs will be recruited after the enrollment of ischemic stroke survivors, at a 1:1 ratio.

#### 4.1.1 Sessions and treatment duration

Healthy volunteers will wear the device alternately on the right and left UL, while for stroke survivors it will be positioned on the paretic limb. All recruited subjects, both HVs and stroke survivors, will undergo a daily session of use of the robotic device for 5 days a week, for 2 weeks, for a total of 10 sessions of application of the device. The duration of the session, if tolerated, is expected to be 20 min. HVs will perform 3 sets of goal-oriented movements, each consisting of 3 attempts for each gesture, each separated by 3 min, while stroke survivors will be asked to attempt to perform the goal-oriented movements. If able to perform the movements, stroke survivors will also perform 3 sets of movements.

#### 4.1.2 Outcome measures

All participants (HVs and stroke survivors) will be administered the following questionnaires (detailed specifically in the subsequent section of this protocol): SUS; UEQ; TARPP-Q (some items). The questionnaires will be completed at three distinct times: after the first session; after the fifth session and at the end of the tenth and last session. To investigate possible presence and distribution of musculoskeletal symptoms in different body regions, the Italian version (20) of Extended Nordic Musculoskeletal Questionnaire (NMQ-E) (21) will be administered. This questionnaire assesses the presence and distribution of musculoskeletal symptoms in 9 body regions: neck, shoulders, upper back, elbows, wrists/hands, lower back, hips/thighs, knees, ankles/feet. It consists of 9 dichotomous questions (yes/no) per body region. The questionnaire will be administered after the first session, and at the end of the last session. At the end of the experimental session, the rehabilitation professionals will be administered the following questionnaires: SUS; UEQ (Table 1).

**Table 1:** Schedule of activities, interventions, and assessments during Phase A of the POWER study.

Activity/assessment	T-1 (screening)	T0 (baseline)	T1 (start session)	T1* (1st session)	T2 (5th session)	T3 (10th session)	Time
Recruitment	X						
Eligibility screening	X						
Informed consent	X						
Clinical data collection		X					
AI data acquisition		X	X	X	X	X	
SUS				X	X	X	
UEQ				X	X	X	
TARPP-Q				X	X	X	
NMQ-E				X		X	
Therapist SUS/UEQ						X	
							End of Phase A

AI, artificial intelligence; SUS, System Usability Scale; UEQ, User Experience Questionnaire; NRS, Numerical Pain Rating Scale; NMQ-E, Extended Nordic Musculoskeletal Questionnaire. *first session.

#### 4.1.3 Data acquisition and purpose of AI algorithms

During Phase A, data from the robotic device will be collected, and the AI algorithms described in the next section of this protocol (Data and measurements).

Phase A will be considered successfully completed when both HVs and stroke survivors provide a positive evaluation of the exosuit across usability, acceptability, wearability, ergonomics, workload, comfort and perceived safety. This condition will be considered positive when the mean SUS scale and UEQ scale score reaches or exceeds 68 and 0.8, respectively, and when no adverse events occur due to device use. The overall SUS score ranges from 0 to 100. Scores ≥ 80 points provide excellent system in terms of usability; 68–80: good usability, and <68 points: problematic usability. Likewise, UEQ scores above 0 indicate a positive experience, while negative scores reflect problems or dissatisfaction.

In the event that the specified cutoff is not reached in even one of the two groups, Phase B will not be initiated and the study will be stopped and redesigned (Figure 1).

All participants involved in the study, both HVs and stroke survivors, must provide informed consent to participate and to the processing of their personal data before enrollment in the study. At the end of the first phase and after verifying the usability and acceptability of the experimental robotic exosuit device, Phase B will be initiated.

### 4.2 Phase B—participants

Phase B will consist of a randomized controlled trial involving subjects with a first-ever ischemic stroke in the subacute phase, consecutively admitted to the Physical Medicine and Rehabilitation of the IRCCS “Casa Sollievo della Sofferenza” after completion of Phase A. Stroke subjects who have already used the device in Phase A will not be eligible for enrollment.

#### 4.2.1 Stroke participants characteristics

Inclusion criteria: (a) adult subjects with first ischemic stroke documented by neuroimaging (brain CT or MRI) with functional impairment of the UL due to -motor deficit: monoparesis/hemiparesis; (b) Stroke survivors with a period from the onset of the ictal event not exceeding 3 weeks; (c) unilateral paresis of the UL; (d) ability to provide informed consent to participate in the study and to the processing of personal data, or presence of an authorized legal representative (e.g., support administrator, guardian, etc.).

Exclusion criteria: (a) bilateral impairment; (b) previous cerebrovascular event or neurological diseases; (c) neglect; (d) inability or unavailability to provide informed consent; (e) neoplastic or other pathologies preventing device application; (f) severe aphasia or communication difficulties; (g) cognitive impairments (score ≤24 on the Mini-Mental State Examination—MMSE); (h) inability to cooperate or behavioral disorders compromising understanding and execution of robotic rehabilitative training;

## 5 Data and measurements

For each stroke participant, demographic, clinical, and rehabilitation treatment-related information will be collected. Specifically, the following information will be collected: demographic data (age, sex, education, family context); time elapsed since stroke onset; type and location of the lesion; neurological severity at treatment start; presence of cognitive disorders; comorbidities (hypertension, diabetes, heart disease, respiratory, urinary and gastro-intestinal diseases).

For all study participants, the wearable robot device will collect the following categories of data: dynamic aspects (applied force); kinematic aspects (gyroscopic data and angles between exoskeleton components).

### 5.1 Purpose of AI algorithms

The AI component of the study will be developed in both study phases but with different objectives.

During Phase A, data acquired from the wearable exosuit while healthy volunteers and stroke participants perform standardized movements will be used to develop the initial AI models. For each required movement, the system will simultaneously collect kinematic and dynamic variables (joint angles, angular velocity, acceleration, applied force, motor torque, and other sensor-derived parameters generated by the wearable device). Movements performed by HVs will constitute the reference dataset representing physiological movement patterns, whereas data collected from stroke participants will be labelled as impaired motor execution.

Using the collected datasets, machine-learning algorithms, for a given movement goal, will be trained to identify characteristic movement patterns, quantify the deviation between patient movements and healthy reference movements, and classify patients according to their movement execution. Based on these analyses and upon assessment from clinicians, the algorithms will estimate the level of robotic assistance required to facilitate movement execution.

Instead, during Phase B, data collected during the rehabilitation sessions, together with the corresponding assessment of participant clinical conditions, will be used to further validate and refine the predictive models. The AI analyses will investigate the relationship between longitudinal changes in sensor-acquired movement features and conventional clinical outcome measures, with the objective of identifying movement characteristics most strongly associated with functional recovery and to estimate the assistance level what would be most appropriate for stroke subjects with similar movement characteristics at different stages of rehabilitation, providing evidence for future adaptive control personalization strategies.

The AI component represents a secondary objective of the study. The predefined rehabilitation protocol and the clinical evaluation of the primary endpoint will not depend on AI outputs. The algorithms will therefore be developed and evaluated independently of the randomized comparison between the intervention and control groups.

## 6 Evaluation and outcome measures

### 6.1 Usability/satisfaction

*System Usability Scale (SUS)*: a standardized tool for evaluating the usability of products, systems, or services. It is a questionnaire consisting of 10 statements, designed to be simple and quick to administer, and widely used in various sectors to measure users’ perception of usability (22). The SUS uses a 5-point Likert scale for each of the 10 statements, with the following response options: strongly disagree; disagree; neutral; agree; strongly agree.

*User Experience Questionnaire (UEQ)*: a standardized tool for measuring the user experience of interactive products such as software, websites, mobile applications, and interactive systems (23). The questionnaire consists of 26 pairs of opposite adjectives exploring six main dimensions of user experience. Items are rated on a 7-point Likert scale, where the score can range from −3 (“totally in favor of the negative term”) to +3 (“totally in favor of the positive term”). The UEQ explores 6 dimensions: *Attractiveness:* Measures the user’s overall impression of the product, including aesthetic and emotional aspects; *Perspicuity*: Assesses the ease of learning and understanding the product; *Efficiency*: Measures the speed and effectiveness of system use; *Dependability*: Examines the subjective feeling of safety and control during use; *Stimulation*: Analyzes the interest and motivation generated by the product. *Novelty*: Evaluates the perceived originality and innovation of the product. Scores are calculated for each dimension as the average of the associated responses.

*Technology Assisted Rehabilitation Patient Perception Questionnaire (TARPP-Q)*: A tool developed to assess patients’ subjective perceptions in technology-assisted rehabilitation programs (24). Responses are provided on a 4-point Likert scale (1 = not at all; 2 = a little; 3 = quite; 4 = very). In the context of this study, items related to three of the four main dimensions will be used: *Positive Attitude, Usability, Discomfort.*

*Italian version of Extended Nordic Musculoskeletal Questionnaire (NMQ-E)* (20): This questionnaire assesses the presence and distribution of musculoskeletal disturbances in 9 body regions.

### 6.2 Neurological assessment

Neurological damage will be assessed in all participants and quantified using the National Institutes of Health Stroke Scale (NIHSS) (25). The Oxford Community Stroke Project classification will be used to assess the type and location of brain damage (26).

### 6.3 Functional measures

Upper limb functional recovery will be assessed according to the International Classification of Functioning, Disability and Health (ICF) model of the World Health Organization, which identifies the components: Body Functions, Activities, and Participation (27). Validated measures widely used in clinical studies for assessing UL functional outcomes will be employed for each ICF component.

- *Primary*: Fugl Meyer Assessment Scale-Upper extremity (FMA-UE).
- *Secondary*: (a) Motricity Index for the upper limb; modified Ashworth Scale, (shoulder adductors, elbow flexors, wrist flexors); Numerical Rating Scale (Body function); (b) Box & Block Test (Activity); (c) Frenchay Activity Index (Participation).

*Fugl-Meyer Assessment Scale (FMA)* (28): The section related to upper limb motor function will be used (FMA-UE) with a score ranging from 0 to 66. The reliability and validity of this scale have been widely demonstrated (29). The minimal clinically important difference (MCID) for FMA-UE is 4.25 points (30).

*Motricity Index (MI)* (31): Items related to the upper limb will be considered, with a score ranging from 0 to 100. The estimated optimal MCID of improvement in the MI arm score is 13 points (32).

*Box & Block Test (B&B)* (33): This test measures gross manual dexterity. The patient is asked to move, one at a time, as many blocks as possible from one compartment to another within 60 s. The standard Minimum Detectable Change (MDC) is roughly 5.5 to 6 per minute (34).

*Modified Ashworth Scale (MAS)* (35): This scale measures muscle spasticity with a score from 0 to 5. It will be used to assess spasticity in the following muscle groups: shoulder adduction, elbow flexion, and wrist flexion.

*Numerical Pain Rating Scale (NPRS)*: This index is a simple, quick, and reliable unidimensional measure to assess pain intensity, similar to the Visual Analog Scale (VAS). The scale ranges from 0 (“no pain”) to 10 (“worst imaginable pain”) (36). Patients are asked to verbally indicate a number from 0 to 10 representing the perceived pain intensity at that moment.

*Frenchay Activity Index (FAI*): Scale for assessing social activity and quality of life after stroke, in relation to upper limb disability, with a total score ranging from 15 to 60 (37). The universally accepted single consensus value for the MCID is not broadly established, but values from 4.25 and 7.25 of 6.7 have been reported (30).

### 6.4 Satisfaction metrics

Only for stroke survivors in experimental group: SUS; TARPP-Q (except dimensions 7 and 8, as they are not applicable to the type of device used in the study) and QUEST 2.0 (dimensions: “Device” and “Most Important Aspects -items 1 to 7”) will be administered at the hospital discharge.

*Quebec User Evaluation of Satisfaction with Assistive Technology (Quest 2.0)* (38): This questionnaire assesses user satisfaction with assistive technology through 12 items divided into two subscales: device (8 items) and service (4 items). Responses are provided on a 5-point Likert scale (1 = not satisfied at all; 5 = very satisfied).

### 6.5 Musculoskeletal symptoms metric

*Italian version* (20) *of Extended Nordic Musculoskeletal Questionnaire (NMQ-E)* (21): To investigate possible presence and distribution of musculoskeletal symptoms in different body regions, the Italian version (20) of Extended Nordic Musculoskeletal Questionnaire (NMQ-E) (21) will be administered at T1 (2 weeks) and T3 (hospital discharge) (Table 2).

**Table 2:** Schedule of participant recruitment, randomization, interventions, and outcome assessments during Phase B of the POWER randomized controlled trial.

Activity/assessment	T-1 (screening)	T0 (baseline)	T1 (2 wks)	T2 (4 wks)	T3 (discharge)	T4 (4 mo/follow-up)
Recruitment	X					
Eligibility screening	X					
Informed consent	X					
Randomization		X				
AI data acquisition		X	X	X	X	X
Neurological assessments						
NIHSS		X				
Oxford/Bamford classification		X				
Functional assessments						
FMA-UE		X	X	X	X	
Motricity Index		X	X	X	X	
MAS		X	X	X	X	
Box & Block Test		X	X	X	X	
NRS (Pain)		X	X	X	X	
FAI						X
Usability/satisfaction						
SUS					X	
TARPP-Q					X	
QUEST 2.0					X	
Musculoskeletal symptoms						
NMQ-E			X		X	

NIHSS, National Institutes of Health Stroke Scale; FMA-UE, Fugl-Meyer Assessment-Upper Extremity; MAS, Modified Ashworth Scale; NRS, Numerical Pain Rating Scale; FAI, Frenchay Activity Index; SUS, System Usability Scale; UEQ, User Experience Questionnaire; TARPP-Q, Technology Assisted Rehabilitation Patient Perception Questionnaire; QUEST 2.0, Quebec User Evaluation of Satisfaction with Assistive Technology; NMQ-E, Extended Nordic Musculoskeletal Questionnaire.

The timeline of all interventions is reported for Phase A and B in Tables 1, 2, respectively.

## 7 Randomization and sample size

Participants meeting the inclusion criteria will be randomly assigned to the control group or the experimental group. A sample randomization approach was followed and a randomization list was computer-generated. The allocation sequence will be concealed from the researcher responsible for enrollment and participant evaluation. Due to the nature of the intervention, blinding of participants and therapists will not feasible. Indeed, the characteristics of the rehabilitative interventions do not allow blinding of patients and therapists involved in the study. Conversely, examiners and data analysts will remain blinded throughout the trial. Considering the FMA-UE as the primary endpoint and data reported by a recent meta-analysis (8), recruiting 29 stroke participants patients per arm ensures 80% statistical power, assuming a type I error of 0.05 to detect a statistically significant and clinically relevant difference of 6 points between the two arms (treatment arm: mean 40, SD 8; control arm: mean 34, SD 8). It is hypothesized that a greater proportion of stroke subjects in the experimental group will achieve a clinically relevant difference of 4.25 points compared to the control group.

### 7.1 Statistical analysis characteristics

Descriptive statistics will be reported as absolute frequencies (absolute values) and percentages for categorical and dichotomous variables. Categorical variables will be compared intra-group and inter-group using the Chi-square test. For continuous variables, the Shapiro–Wilk normality test will be performed to assess distribution. Normally distributed data will be expressed as mean ± standard deviation (SD). Non-normally distributed data will be described using median and interquartile range (IQR). Repeated measures ANOVA will be used for intra-group and inter-group comparisons for normally distributed variables, and the non-parametric Friedman test for repeated measures will be used for non-normally distributed variables. Balance between arms will be verified computing the Standardized Mean Differences. If any potential confounders would result unbalanced between arms, an ANCOVA model will be applied adjusting for the unbalanced confounders. A *p*-value <0.05 will be considered as statistically significant. All statistical analyses will be performed using the software R (version 4.6.0).

## 8 Rehabilitative intervention

The rehabilitative intervention will focus on the functional recovery of the UL, with a specific focus on reaching and grasping movements, which are essential factors for functional outcome.

### 8.1 Experimental group

Patients allocated to the experimental arm will receive upper-limb rehabilitation supported by the wearable elbow exosuit, designed to assist elbow flexion and extension. Because elbow mobilization plays a central role in functional reaching and object-interaction tasks, the intervention will focus on restoring these movements through structured, goal-oriented training. Although the robotic device provides assistance specifically for elbow flexion and extension, these joint-level movements will be embedded within functional, task-oriented activities.

### 8.2 Control group

Stroke participants in the control group will undergo traditional rehabilitative intervention for UL recovery, based on neurodevelopmental techniques and proprioceptive neuromuscular facilitation.

### 8.3 Rehabilitation duration

All stroke participants will undergo 45 min of rehabilitation per day, 5 days a week, for 6 weeks with the wearable robotic device and traditional rehabilitation for experimental and control groups, respectively. All participants will therefore receive 30 rehabilitation treatment sessions. Subsequently, all participants in both groups will continue with traditional rehabilitation. The number of sessions actually performed by participants will be recorded, as well as the reasons that prevented the execution of the rehabilitative treatment. No concurrent rehabilitation was permitted.

## 9 Timing of functional evaluation

All enrolled stroke subjects will undergo functional evaluation at baseline (T0), after 2 weeks (T1), after 4 weeks (T2), at hospital discharge (T3). All measurements will be administered at the above time points, except for the Frenchay Activity Index, which will be administered at the 4-month follow-up.

## 10 Device used in the study

The prototype device is a hybrid, non-invasive wearable exosuit for arm assistance (flexion and extension movements) developed by the Institute of Mechanical Intelligence of the Scuola Superiore Sant’Anna in Pisa (39–41) (Figure 2). It is a unique prototype specimen. The device operates at very low voltage (<50 V according to Directive 2014/35/EU) and is equipped with parts actuated by electronically controlled DC electromagnetic motors, not directly applied to the user (forces are exchanged through sheathed tendons), and a Wi-Fi communication system (standard 802.11). The wearable part of the device consists of five modules: *Back module,* containing the control unit and actuation group; *Shoulder module*, consisting of a padded fabric shoulder orthosis; *Arm module*, consisting of an ergonomically shaped ABS plastic support with thicknesses allowing it to easily fit arms of different sizes; *Forearm module*, consisting of an ergonomically shaped ABS plastic support with thicknesses allowing it to easily fit arms of different sizes; *Hand module*, composed of a fabric glove and two length-adjustable fabric straps. The overall weight of the device is 4 Kg.

**Figure 2:**
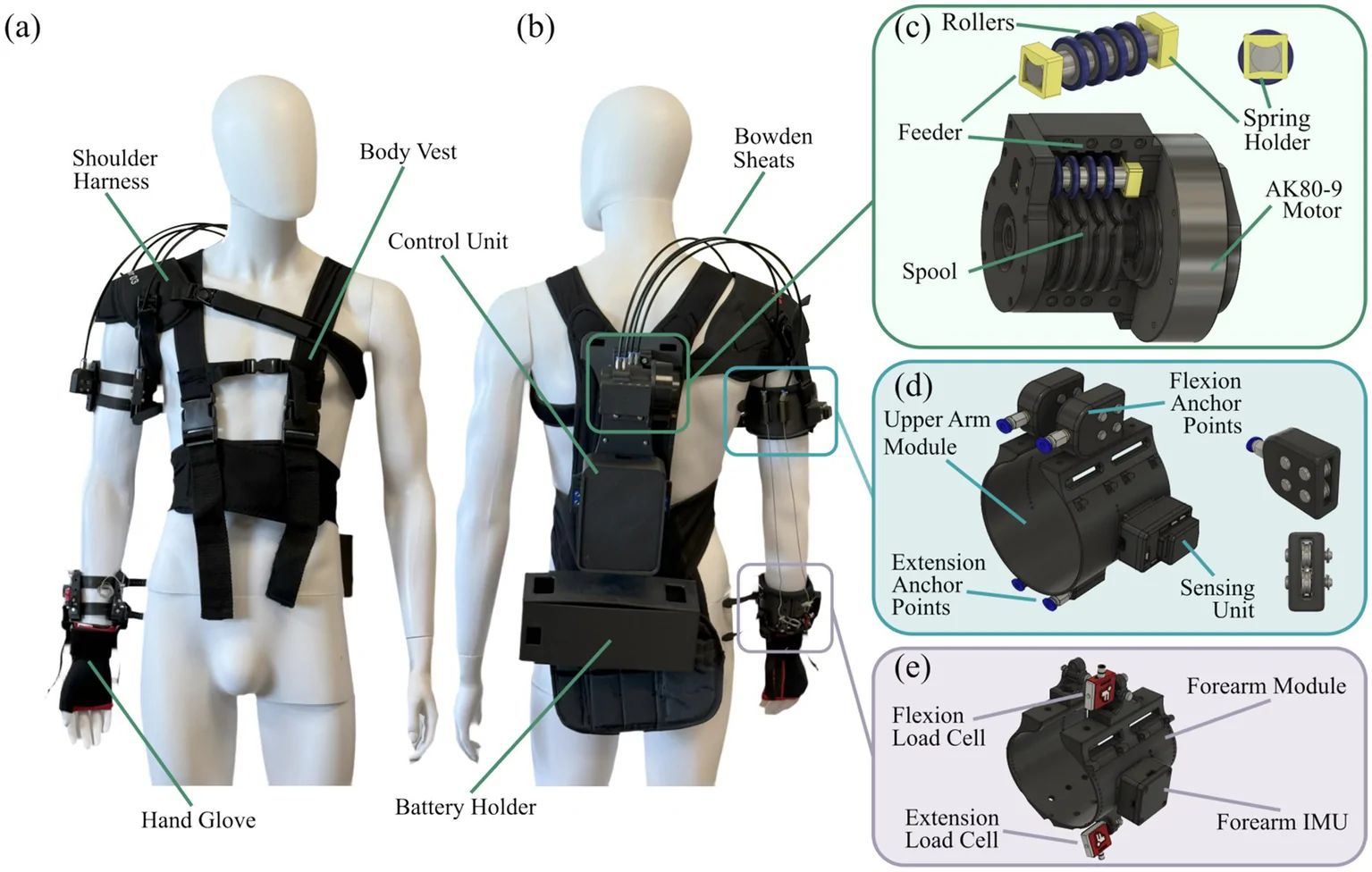
Robot device exosuit: **(a)** Front view of the exosuit showcasing its wearable structure; **(b)** back view of the exosuit highlighting the backpack-mounted actuation unit and harness system; **(c)** detailed mechanical design of the actuation unit and the passive cable feeder mechanism for tension maintenance; **(d)** upper-arm cuff with integrated guide mechanism ensuring smooth Bowden cable routing; **(e)** forearm cuff design with ergonomic features and sensor integration.

## 11 Risk analysis and adverse effects

The risk analysis conducted by the manufacturer highlighted that the probability of a failure occurring and the extent of the resulting damage are very low. During the trial, the device will be worn by subjects in a seated position and under the supervision of a physiotherapist who will be next to the participant during all study activities. Considering the weight of the device and the possible comorbidities of stroke participants, all enrolled subjects, after wearing the exosuit, will undergo ECG and blood pressure monitoring to detect potential cardiovascular adverse events. All safety-related adverse events occurring during the study period (in both Phase A and Phase B) will be monitored and recorded by therapists and/or other healthcare professionals. During interventions or evaluations, the therapist will monitor and note any side effects or discomfort caused by the use of the exoskeleton, including: hemodynamic and cardiovascular alterations (hypertension, hypotension, arrhythmias); fatigue, joint pain, skin pressure, blisters or other forms of skin, muscle, or joint discomfort.

The intervention or evaluation will be stopped immediately if any of the above symptoms occur. At the end and during each intervention or evaluation session, the therapist will check and note the possible onset of further side effects or discomfort, including dizziness, malaise, headache, nausea, fainting, dyspnea, or any other complaints reported by the participant.

## 12 Data usage

In this study, the collected data will be classified into two categories.

*Clinical data and telemetry data*. Researchers responsible for data collection will also handle data entry in a blinded manner. Participants’ personal data will be processed in compliance with Legislative Decree 30/06/2003 No. 196 (Personal Data Protection Code) and the Regulation (EU) 2016/679 of the European Parliament (GDPR) and of the Council of 27 April 2016^1^ on the protection of natural persons with regard to the processing of personal data. These data will be recorded anonymously using an alphanumeric code, stored electronically. Clinical data will be manually entered into a dedicated digital platform.

Telemetry data will be automatically acquired in real time by the robotic device through integrated sensors, which will send information to the digital platform via appropriate communication protocols. These data will include parameters such as speed, motor torque, and other elements useful for evaluating the participant’s action during upper limb motor activation.

All data will be associated with anonymous codes and organized in a database, ensuring that only the attending physician can access personal health information, timestamps, and the type of movement performed during sessions. The list linking codes to participant identities will be stored on a secure digital platform accessible exclusively to the study’s principal investigator. Access to clinical data will be restricted to authorized personnel, while telemetry data will only be viewable in anonymous form. All data will be pseudonymized and the resulting dataset will be transmitted to a purposely developed cloud server through a secure connection. The cloud service is designed by Exprivia, a company that operates in the digital health sector and is collaborating on the study. The primary purpose of data processing will be statistical analysis for research purposes.

### 12.1 Data processing objectives

*Training the Anomaly Detection Algorithm*: Telemetry and participant data will be collected and used to train, optimize, and test an algorithm designed to identify anomalies in stroke survivors movements.

*Comparative Analysis*: The algorithm calculates the discrepancy between the target movement (derived from healthy subjects) and the patient’s actual movement.

## 13 Discussion and conclusion

Robotic rehabilitation (RR) intervention in stroke survivors has rapidly developed over the last 2 decades and this approach has been shown to be effective as traditional rehabilitation to improve neurological impairment and disability (8). A number of robotic devices have been used for the rehabilitation of the paretic UL in post-stroke subjects, which have demonstrated to be effective. Despite these encouraging results, several unresolved issues remain, including optimal timing after stroke, training intensity and duration, and—most importantly—the translation of motor gains into meaningful functional improvements and participation in daily life. Measures commonly used to assess UL recovery can detect improvements in muscle strength and movement, but may not capture specific aspects of function such as dexterity, movement fluency, and the impact on daily activities and participation. To overcome these limitations of commonly used measures, indicators such as clinical significance and MCID have been used. In this respect, Verola et al. (42) conducted a meta-analysis of the effects of robotic rehabilitation for UL after stroke on clinical relevance, but the study did not find significant differences when RR was compared with other strategies. Furthermore, RR had no significant effects on UL capacity considered as the subjects’ potential in transporting, moving and handling objects and in the fine use of the arm, in the expression of activity and participation according to the domains coded in the ICF (27). This aspect is very important in the rehabilitation process because, correct motor skills are necessary to perform the numerous and complex motor activities of self-care and daily living (43–45).

One of the main barriers to the widespread adoption of robotic rehabilitation lies in the design and usability of currently available devices. Most UL robotic systems are fixed, bulky, and restricted to specialized rehabilitation facilities. Therapists and patients frequently report concerns related to complexity, setup time, discomfort, and limited adaptability to functional tasks, all of which negatively affect acceptance and real-world applicability (10). In this regard, technological development could promote the production of soft, wearable devices to overcome some of the limitation of currently used robotic equipment (11, 12). Studies have shown that portable, lightweight devices can be used to improve range of motion (46) and synergy of impaired arm flexor muscles (14). Indeed, some robotic devices have been designed to be portable, and their effect on improving impaired UL has been described in a recent meta-analysis (47). The aim of the study was to identify portable robots that could be operated by an average adult. All the robotic machines were used in hospital, and considering their wearability or portability, three weighed 46.4 kg and one 15 kg, while data for the remaining devices were not reported. The authors stated that the term “portable” was relative due to the lack of a clear definition, and that portability was primarily seen in terms of the ease of transporting the robot to the patient’s home. Therefore, even if labeled as “portable” often the robotic devices remained heavy and poorly manageable, limiting their use outside institutional settings. Conversely, the wearable exoskeleton evaluated in the present protocol is specifically designed to overcome these limitations. With a total weight of 4 kg and a soft, modular, non-invasive structure, the device represents a substantial advancement compared with existing robotic systems. Its design aims to facilitate donning and doffing, improve comfort, and allow use in seated functional tasks, potentially extending rehabilitation beyond conventional clinical environments. Importantly, this study explicitly addresses usability, acceptability, and safety through a dedicated feasibility phase (Phase A), an aspect that has been largely neglected in previous research on wearable robotic devices.

Another important aspect is the growing use of machine learning and AI to support robotic rehabilitation. Artificial intelligence is revolutionizing all fields of activity and the human sciences. Its applications in the medical field are growing, from facilitating data classification and analysis to event risk prediction, prognostic trajectories, optimization of therapeutic interventions (48, 49) and care delivery (50). In the field of rehabilitation, particularly in patients with neurological conditions such as Parkinson’s disease and stroke, AI has been tested in intervention, prognosis, assessment, and monitoring (15–17). AI could facilitate the identification of prognostic factors and rehabilitation strategies important for functional recovery (51). In this regard, AI is increasingly associated with RR in improving patient recovery and paves the way for therapeutic practices (52). Motor gestures are the result of complex neurophysiological mechanisms and neural networks, whose mechanisms are only partially understood. AI could be highly effective in predicting movement intentions and detecting compensatory movements, but several pitfalls, such as potential model explainability issues and poor generalization ability when used outside of the institutional context, have not been resolved (53). Our study aims to identify, using AI algorithms, which dynamic and kinematic muscle components such as force, acceleration, velocity, motor torque, and other elements supporting goal-directed gestures can predict recovery, providing insights for future personalized rehabilitation interventions and clinical decision-making. This objective is in line with a recent study aimed at building predictors of clinical outcome that were based, in that case, on the measurement of robot-measured features in subacute stoke survivors (54). Furthermore, the positive effect of exosuit could facilitate patients’ home rehabilitation and stimulate the paretic arm in the execution of functional activities and gestures in a real-life context.

If feasibility and effectiveness are confirmed, the new robotic device evaluated in this study could represent a meaningful step toward more personalized, accessible, and functionally oriented rehabilitation pathways. In the long term, such device may support continuity of care beyond inpatient settings, promote active use of the paretic arm in daily life, and facilitate home-based rehabilitation programs. Ultimately, the integration of wearable robotic and AI-driven analytics has the potential to reshape post-stroke rehabilitation by moving from impairment-focused interventions toward functionally relevant, patient-centered, and data-informed therapeutic strategies.

## Data Availability

The original contributions presented in the study are included in the article/supplementary material, further inquiries can be directed to the corresponding author/s.

## Footnotes

1
https://www.garanteprivacy.it/documents/10160/0/Codice+in+materia+di+protezione+dei+dati+personali+%28Testo+coordinato%29
